# Efficacy of intraoperative recurrent laryngeal neuromonitoring during surgery for esophageal cancer

**DOI:** 10.1002/ags3.12394

**Published:** 2020-09-17

**Authors:** Shigeru Takeda, Michihisa Iida, Shinsuke Kanekiyo, Mitsuo Nishiyama, Yukio Tokumitsu, Yoshitaro Shindo, Shin Yoshida, Nobuaki Suzuki, Shigefumi Yoshino, Hiroaki Nagano

**Affiliations:** ^1^ Department of Gastroenterological, Breast and Endocrine Surgery Yamaguchi University Graduate School of Medicine Yamaguchi Japan; ^2^ Oncology Center Yamaguchi University Hospital Yamaguchi Japan

**Keywords:** esophageal cancer, esophagectomy, intraoperative neurophysiological monitoring, minimally invasive surgery, recurrent laryngeal nerve palsy

## Abstract

**Aim:**

To evaluate the efficacy of intraoperative neuromonitoring in identifying recurrent laryngeal nerves and decreasing the incidence of nerve injury in minimally invasive esophagectomies for esophageal cancers.

**Methods:**

A total of 167 minimally invasive esophagectomy patients were retrospectively reviewed. They were divided into intraoperative neuromonitoring (n = 84) and no intraoperative neuromonitoring (n = 83) groups, based on whether or not intraoperative neuromonitoring was used during surgery. We compared short‐term surgical outcomes and incidence of recurrent laryngeal nerve palsy between the two groups before and after propensity score matching. The association between the loss of signal and recurrent laryngeal nerve palsy was also evaluated.

**Results:**

The incidence of recurrent laryngeal nerve palsy (grade 2 and higher) was lower in the intraoperative neuromonitoring group than in the no intraoperative neuromonitoring group (6.0% vs 21.2%, *P* = 0.02). The rate of recurrent laryngeal nerve palsy recovery within 6 months was also significantly higher in the intraoperative neuromonitoring group (87.5% vs 20.0%, *P* < 0.01). The positive and negative predictive values of intraoperative neuromonitoring for recurrent laryngeal nerve palsy were 60% (9/15) and 86.9% (60/69), respectively. The duration from paralysis to recovery was shorter in recurrent laryngeal nerve palsy cases with negative loss of signal results than in cases with positive loss of signal results (median: 43 days vs 95 days).

**Conclusion:**

Intraoperative neuromonitoring is useful in identifying recurrent laryngeal nerves and may aid in reducing the incidence of recurrent laryngeal nerve injury during esophageal surgery.

## Graphical abstract

Intraoperative neuromonitoring was useful in reducing the incidence of laryngeal nerve palsy associated with esophageal surgery.

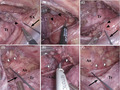

## 1 INTRODUCTION

Esophageal cancer is associated with a high incidence of lymph node metastasis and often requires the use of esophagectomy with radical lymphadenectomy.^1^ In recent years, owing to the development of endoscopic equipment and progress in surgical techniques, minimally invasive esophagectomy (MIE) has become the treatment of choice in patients with esophageal cancer with locoregional lymph node metastasis. The advantages of MIE over conventional open transthoracic esophagectomy have been reported in several studies.^2^, ^3^, ^4^, ^5^ However, an increased frequency of postoperative complications such as recurrent laryngeal nerve palsy (RLNP) has been observed; therefore, care must be taken during surgery.^6^ Although delicate surgical procedures can now be performed owing to the enlarged view offered by endoscopy, the recurrent nerve is vulnerable to injury due to thermal damage or towing, and this could cause postoperative vocal cord palsy and swallowing dysfunction. Furthermore, as postoperative complications affect patients’ long‐term prognoses,^7^ preventing RLNP is very important.

Intraoperative neuromonitoring (IONM) has gained widespread acceptance as a useful tool for the preservation of nerve function during thyroid surgery and recently during surgery for esophageal. Few studies have focused on the importance of IONM in surgeries for esophageal cancer, and its usefulness has not been revealed.

Therefore, in this study, we aim to evaluate the efficacy of IONM in identifying RLN injury and reducing the incidence of RLNP.

## 2 PATIENTS AND METHODS

### 2.1 Patients

A total of 228 consecutive patients underwent esophagectomy for esophageal cancer at the Yamaguchi University Hospital (Yamaguchi, Japan) between 2009 and 2018. Out of these, 167 patients underwent MIE in the prone position and their treatment outcomes were retrospectively analyzed in this study. The inclusion criteria for MIE were patients with the Union for International Cancer Control (UICC) clinical stage I–IVA, except for the patients with T4b or preoperative radiation therapy. In 2015, our institute introduced IONM for all esophagectomies. The patients in this study were divided into two groups: the IONM group (n = 83), comprising patients who had surgery with neuromonitoring between 2015 and 2018, and the no IONM group (n = 84), comprising patients who had conventional surgery without neuromonitoring between 2009 and 2014. We compared the short‐term surgical outcomes and the incidence of RLNP between both groups.

### 2.2 Data collection

Preoperative tumor stage evaluations included: a medical interview; physical examination; upper gastrointestinal endoscopy and biopsy; computed tomography scan of the chest, abdomen, and pelvis; positron emission tomography‐computed tomography; and blood tests. Initial evaluation revealed that none of the patients had RLN paresis or paralysis before surgery.

Clinical data of all patients were collected from a database at our institution. Tumor staging was done according to the UICC esophageal cancer TNM staging system (8th edition). For postoperative complications, RLNP was divided into grades 1 and 2 or higher using the Clavien‐Dindo (CD) classification. RLNP requiring the use of antibiotics for the treatment of aspiration pneumonia was classified as grade 2. Aspiration pneumonia was defined as follows: (a) pneumonia following a witnessed macroaspiration event, in which case the content of aspiration was confirmed in the trachea; (b) a swallowing video endoscopy‐confirmed aspiration and repeated symptoms of aspiration; (c) a computed tomography scan showing infiltrative findings in the superior or basal segment of the lower lobes, or the posterior segment of the upper lobes of the lungs.

### 2.3 Surgical procedure

**FIGURE 1:**
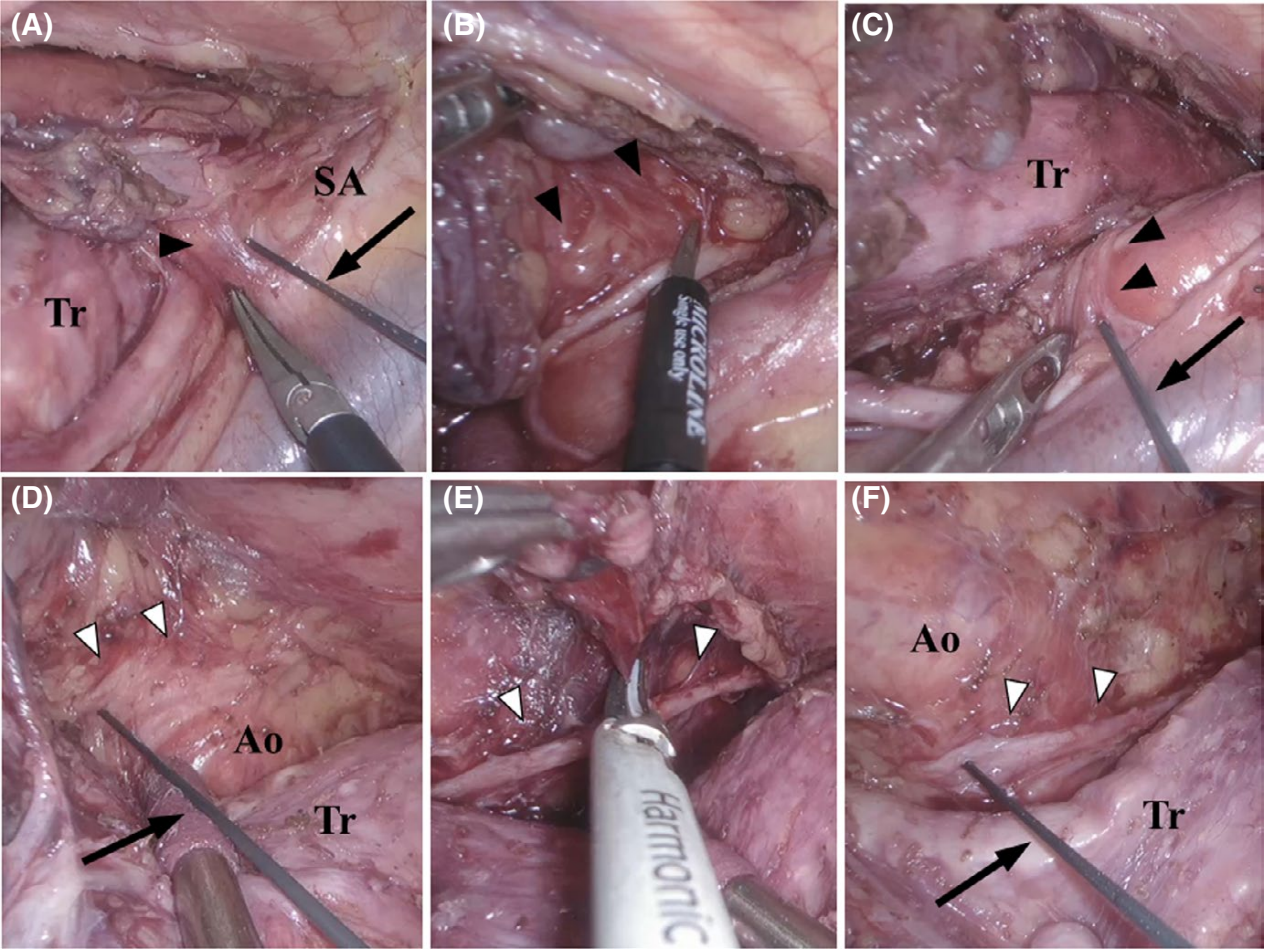
Dissection of recurrent laryngeal nerve lymph node. A, The Right RLN was identified with the neural stimulation; black arrowhead, right recurrent laryngeal nerve; black arrow, stimulator probe; SA, right subclavian artery; Tr, trachea; RLN, recurrent laryngeal nerve. B, The branches from the RLN were divided with the scissors or the ultrasonic scalpel; black arrowhead, right RLN. C, The intermittent nerve stimulation at the intersection of the right subclavian artery was performed to observed response of EMG activity; black arrowhead, right recurrent laryngeal nerve; black arrow, stimulator probe; Tr, trachea. D, The left RLN was identified with the neural stimulation under the aortic arch; white arrowhead, left recurrent laryngeal nerve; black arrow, stimulator probe; Ao, aortic arch; Tr, trachea. E, The connective tissue and vessels were dissected with the ultrasonic scalpel preserving RLN; white arrowhead, left RLN. F, The intermittent nerve stimulation at the intersection of the aortic arch was performed to observed response of EMG activity; white arrowhead, left recurrent laryngeal nerve; black arrow, stimulator probe; Ao, aortic arch; Tr, trachea

We performed three‐field esophagectomy with an anastomosis in the neck and started with the thoracic component. Right thoracoscopic access was secured, and four trocars were inserted. A transitory CO_2_ pneumothorax (6 mm Hg) was instituted in the prone position, followed by esophageal mobilization and mediastinal lymphadenectomy. The lymph nodes around the RLN were dissected using an ultrasonic scalpel and cold cutting scissors (Figure 1).

A gastric conduit was constructed and perigastric lymphadenectomy was performed in the supine position along with hand‐assisted laparoscopic surgery. Through a collar‐shaped neck incision, both RLNs were identified before the removal of the cervical paraesophageal nodes. For patients with mediastinal and perigastric lymphadenectomy as D2 dissection, right cervical paraoesophageal node dissection was completed during the transthoracic procedure. In D3 lymph node dissection, both supraclavicular lymphadenectomy was performed through the neck incision in addition to D2 dissection. The gastric conduit was pulled through the posterior mediastinum or retrosternal routes, and cervical esophagogastric anastomosis was performed using an end‐to‐side circular stapler.

Three surgeons performed the surgeries. The efficiency of each surgeon was classified based on the number of procedures they had performed (either ≥ 31 or ≤30).

### 2.4 IONM

In IONM, the evoked electromyogram (EMG) of a muscle is monitored by direct electrical stimulation of its innervating nerve with a nerve stimulation probe during surgery after an electrode is set on the muscle. Neural function was confirmed by the sound converted from the EMG response.

The patients in the IONM group were intubated under general anesthesia using the NIM TriVantageTM EMG tube (Medtronic ENT) and underwent intraoperative RLN monitoring by the NIM‐response® system (Medtronic ENT) for EMG. Neural stimulation was done with a hand‐held monopolar stimulator (Medtronic ENT), usually with currents of 1.0 mA. When stimulation of the RLN and the vagus nerve produced no EMG activity (electrical silence) or substantially reduced EMG activity (<100 mV), loss of signal (LOS) was confirmed, and neural injury was suspected.

In the intrathoracic procedure, before the RLN was fully exposed and visualized, the IONM probe was used to stimulate the cords in order to search for and identify RLN (Figure 1A,D). EMG activity was observed in response to intermittent nerve stimulation at RLN branched off the vagus nerve at the right subclavian artery and at the aortic arch (Figure 1C,F). If LOS was detected, distal points were stimulated for the identification of the site of nerve conduction impairment. Lastly, before the end of surgery, the cervical vagus nerve was stimulated to observe neural activity.

We reviewed the intraoperative video files of all patients with RLNP in order to identify the likely site of damage and verify the procedure that caused it.

### 2.5 Examination of vocal cord movement

RLNP was confirmed seven days after the surgery using indirect laryngoscopy. An otolaryngologist observed the movements of patients’ vocal cords to check for paralysis. RLNP was defined as fixation and disturbance of vocal cord mobility and the affected side was noted. Patients with RLNP were followed‐up and re‐examined using laryngoscopy every 1‐3 months until the palsy improved. Recovery from RLNP was defined as complete improvement in the mobility of the affected vocal cord, after which follow‐up with the otolaryngologist was deemed complete. Vocal cord palsy failing to resolve within 12 months was considered to be a permanent RLNP.

### 2.6 Statistical analysis

All statistical analyses were performed using the SPSS Statistics version 25.0 software (SPSS Inc, Chicago, IL, USA). We reported categorical variables as absolute and percentage values and continuous variables as medians and ranges. Comparisons between the frequencies of the categorical variables were assessed using the Pearson's chi‐square test or Fisher's exact test, and were corrected for continuity. Comparisons between the median of continuous variables were assessed using the Mann‐Whitney *U* test. Multivariate logistic regression analysis was performed to determine the risk factors of RLNP. Propensity scores were calculated by using a multivariable logistic regression model adjusted for age, sex, Brinkman index, respiratory function (%VC, %FEV), Albumin, liver function (ICG15), renal function (24 hour CCr), diabetes mellitus, location of tumor, tumor depth (cT), node status (cN), node status around RLN, prior therapy, surgeon's experience, and extent of lymph node dissection. For all matching, we performed one‐to‐one nearest‐neighbor matching with replacement using a caliper of 0.2. Covariate balance of the matched cohort was assessed using the absolute standardized differences, with less than 0.1 taken to indicate good balance. Cumulative recovery rates were identified with the Kaplan‐Meier method and were used to describe the recovery from RLNP in the different patient groups. Log‐rank statistics were used for comparisons between the groups. *P*‐values were derived from the two‐tailed tests, and a *P*‐value < 0.05 was considered statistically significant.

## 3 RESULT

### 3.1 Clinical and surgical findings

A total of 167 patients were included in our study. Among these patients, 83 underwent MIE without IONM (no IONM group) and 84 underwent MIE with IONM (IONM group). Table 1 shows the demographic and clinical profiles of the patients in both groups. No significant differences in sex, age, smoking history index, pulmonary function, prevalence of diabetes, serum albumin, and liver function were noted between the two groups. Cancer characteristics (location of tumor, depth of tumor invasion, and metastasis to regional nodes and nodes around the RLN) were also not significantly different. Differences were observed in terms of renal function, prior therapy, surgeon's experience, and extent of node dissection (*P* < 0.01). The patients’ surgery‐related characteristics are shown in Table 2. The intrathoracic surgery time in the IONM group was shorter than that in the no IONM group (331 minutes vs 3307 minutes, *P* = 0.01). There was less blood loss in the IONM group when compared to the degree of blood loss in the no IONM group (237 mL vs 341 mL, *P* < 0.01). There was no difference in the number of resected thoracic lymph nodes and upper mediastinal lymph nodes between the two groups. The durations of intensive care unit stay, hospital stay, and postoperative fasting were similar in both groups.

**Table 1:** Patient characteristics

Factors		Before matching				After matching			
		no IONM (n = 83)	IONM (n = 84)	*P*‐value	Std diff	no IONM (n = 47)	IONM (n = 47)	*P*‐value	Std diff
Age		66 (47‐81)	68 (45‐86)	0.20	0.152	68 (54‐81)	67 (47‐80)	0.33	0.061
Sex	Male/Female	72 /11	70 /14	0.53	0.095	41/6	40/7	0.76	0.043
Brinkman index		600 (0‐880)	600 (0‐5200)	0.77	0.068	750 (0‐2880)	700 (0‐5200)	0.93	0.047
%VC	<80/≤80	2/81	3/1	1.00	0.091	1/46	1/6	1.00	0.000
%FEV1.0	<70/≤70	33/50	32/2	0.875	0.034	18/29	19/28	0.83	0.043
Albumin	g/dL	4.2 (3.0‐5.0)	4.2 (2.7#x02010;4.9)	0.06	0.299	4.1 (3.0‐5.0)	4.2 (2.7‐4.9)	0.78	0.057
ICG15R	%	8.9 (0.7‐33.8)	10.0 (0.9‐31.9)	0.17	0.051	8.9 (2.9‐33.8)	9.1 (0.9‐21.0)	0.17	0.021
24hCCr	L/day	114.7 (46.9‐191.4)	87.9 (38.6‐211.3)	<0.01*, a	0.711	106.9(46.9‐154.0)	100.0 (49.6‐211.0.3)	0.60	0.035
DM	Yes/No	8/75	9/75	0.81	0.035	8/39	7/40	0.77	0.058
Location of tumor	U/M/L	16/41/26	21/38/25	0.601	0.138	11/20/16	11/20/16	1.00	0.000
cT(UICC 8th) ^a^	T1/2/3	47/17/19	40/14/30	0.137	0.181	23/11/13	23/6/18	0.33	0.000
cN(UICC 8th) ^a^	N0/1/2/3	49/26/4/3	46/21/14/3	0.127	0.086	23/18/3/3	24/12/10/1	0.11	0.042
cN around RLN	Yes/No	22/61	23/61	0.96	0.019	17/30	15/32	0.66	0.089
Prior therapy	Yes/No	30/53	49/35	<0.01**, a	0.455	27/20	26/21	0.83	0.042
Surgeons' experience	0‐30/30 over	50/33	24/60	<0.01**, a	0.672	18/29	20/27	0.67	0.086
Extent of lymph node dissection	D2/D3	52/31	30/54	<0.01**, a	0.559	22/25	20/27	0.67	0.085

Note

Std diff less than 0.1 suggests adequate variable balance after propensity score matching.

Abbreviations: 24hCCr, 24‐hour urine creatinine clearance; D2, mediastinal and perigastric lymphadenectomy; D3, 2 fields + cervical lymphadenectomy; DM, diabetes mellitus; FEV1.0, percentage forced expiratory volume in one second; ICG15R, Indocyanine green retention rate after 15 minutes; IONM, intraoperative neuromonitoring; L, lower third; M, middle third; Std Diff, standardized differences; U, upper third; VC, percentage vital capacity.

^a^ Union for International Cancer Control 8th edition;

* *P*‐value for two groups compared using the Mann–Whitney *U* test; significance was set at a value lower than 0.05.

** *P*‐value for two groups compared using the chi‐square test; significance was set at a value lower than 0.05.

**Table 2:** Surgical outcomes

Factors		Before matching			After matching		
		No IONM (n = 83)	IONM (n = 84)	*P*‐value	No IONM (n = 47)	IONM (n = 47)	*P*‐value
Operation time	min	536 (422‐860)	552 (355‐804)	0.79	545 (439‐860)	566 (355‐804)	0.43
Thoracic time	min	331 (260‐430)	307 (178‐525)	0.01*	331 (265‐430)	324 (178‐525)	0.38
Bleeding	mL	240 (30‐1630)	180 (40‐163)	<0.01*	280 (30‐1630)	180 (49‐1100)	0.01*
Number of thoracic LNs		25.5 (6‐50)	27 (10‐59)	0.26	26.5 (9‐50)	28 (13‐59)	0.7
Number of upper mediastinal LNs		11 (1‐30)	10 (1‐30)	0.57	11 (1‐32)	11 (2‐30)	0.85
pT (UICC 8th)^*^, ^a^	pT 0/is/ 1 /2 /3	2/52/10/19	1/48/12/22	0.71	2 /26 /5 /14	0/ 27 /8 /12	0.5
pN (UICC 8th)^*^, ^a^	pN 0 /1 /2/ 3	43/23/9/8	47/16/17/4	0.15	21/12/7/7	21/10/13/3	0.31
pStage (UICC 8th)^*^, ^a^	pS 0 /1/ 2 /3 /4	1/35/17/20/10	2/31/22/19/10	0.97	1/18/5/14/9	0/17/9/14/7	0.83
ICU stay	day	3 (2‐21)	3 (1‐16)	0.08	3 (2‐21)	3 (1‐16)	0.86
Fasting period	day	8 (7‐90)	8 (7‐50)	0.07	8 (7‐90)	8 (7‐50)	0.01*
Hospital stay	day	28 (15‐256)	29 (10‐180)	0.95	28 (15‐256)	28 (0‐180)	0.23

Abbreviations: IONM, intraoperative neuromonitoring; LN, lymph node; pStage, pathology stage; ICU, intensive care unit

^a^ Union for International Cancer Control 8th edition.

* *P*‐value for two groups compared using the Mann–Whitney *U* test; significance was set at a value lower than 0.05.

### 3.2 Postoperative morbidity

There was no difference in the overall RLNP incidence rate between the before matched groups (26.5% vs 21.2%, *P* = 0.44); however, the number of CD grade 2 or higher RLNP cases was lower in the IONM group than in the no IONM group (5.9% vs 15.6%, *P* = 0.04) (Table 3). There was no difference between the groups in terms of the presence of pneumonia, anastomotic leakage, and other complications. The postoperative 30‐day mortality rate did not differ significantly between the two groups. One patient in the IONM group died from necrotizing soft tissue infection with sepsis.

**Table 3:** Morbidity and mortality

Factors	Before matching					After matching				
	No IONM (n = 83)		IONM (n = 84)		*P*‐value	No IONM (n = 47)		IONM (n = 47)		*P*‐value
*Morbidity*										
Overall RLNP	22	26.5%	18	21.4%	0.44	15	31.9%	8	17.0%	0.09*
(Grade 1/ 2/ 3a/ 3b /4a) ^**^, ^a^	(9/7/3/3/1)		(13/4/0/1/0)			(5/5/3/1/1)		(6/2/0/0/0)		
(Right/ Left/Bilateral)	(1/18/3)		(1/16/1)			(0/11/2)		(0/8/0)		
RLNP (≥Grade 2)^**^, ^a^	13	15.6%	5	5.9%	0.04*	10	21.2%	2	6.0%	0.02**
(Right/ Left/Bilateral)	(1/9/3)	(0/4/1)				(1/7/3)		(0/2/0)		
Pneumonia	19	22.8%	21	25.0%	0.3	9	19.1%	11	23.4%	0.8
(Grade2/ 3a/ 3b /4a)^**^, ^a^	(8/4/3/4)		(14/5/0/2)			(4 /2/0/3)		(7/2/0/2)		
Anastomotic leakage	10	12.0%	15	17.8%	0.41	7	14.8%	8	17.0%	0.77
(Grade2/ 3a/ 3b /4a)^**^, ^a^	(1/6/2/1)		(2/12/1/0)			(1/3/2/1)		(2/5/1/1)		
Surgical site infection	12	14.4%	18	21.4%	0.24	7	14.8%	10	21.2%	0.42
(Grade1 /2 /3a /5)^**^, ^a^	(2/3/7/0)		(2/4/11/1)			(1/2/0 /4/0)		(1/2/1/5/1)		
Chylothorax	3	3.6%	2	2.3%	0.83	2	4.2%	0	0.0%	0.49
(Grade2 /3b)^**^, ^a^	(1/2)	(1/1)				(0/2)				
Postoperative hemorrhage	3	3.6%	1	1.1%	0.3	1	2.1%	1	2.1%	1.00
(Grade3b)^**^, ^a^	(3)		(1)							
Trachial fistula	1	1.2%	1	1.1%	0.99	1	2.1%	1	2.1%	1.00
(Grade3a /3b)^**^, ^a^	(1/0)		(0/1)			(1/0)		(0/1)		
Mortality	0	0.0%	1		0.31	0	0.0%	0	0.0%	‐

Abbreviations: IONM, intraoperative neuromonitoring; RLNP, recurrent laryngeal nerve palsy; SSI, surgical site infection.

^a^ Clavien‐Dindo classification

* *P*‐value for two groups compared using the chi‐square test; significance was set at a value lower than 0.05.

** *P*‐value for two groups compared using the Fisher's exact test; significance was set at a value lower than 0.05.

The number of patients that recovered completely form RLNP are shown in Table 4. Recovery from RLNP was greater in the patients of the IONM group than in the patients in the no IONM group (94.4% vs 27.7%, *P* < 0.01). Similarly, a larger proportion of patients in the IONM group compared to those in the no IONM group recovered from RLNP within 6 months (88.8% vs 22.7%, *P* < 0.01).

**Table 4:** Values of recovery from RLNP

Factors	Before matching					After matching				
	No IONM (n = 22)		IONM (n = 18)		*P*‐value	No IONM (n = 15)		IONM (n = 8)		*P*‐value
Recovery from RLNP	6	27.7%	17	94.4%	<0.01*	5	33.3%	7	87.5%	0.02*
Recovery within 6 months	5	22.7%	16	88.8%	<0.01*	3	20.0%	7	87.5%	<0.01*

Abbreviations: IONM, intraoperative neuromonitoring; RLNP recurrent laryngeal nerve palsy.

* *P*‐value for two groups compared using the Fisher's exact test; significance was set at a value lower than 0.05.

Univariate and multivariate analysis of the risk factors for RLNP with CD grade 2 or higher are shown in Table 5. Multivariate analysis also showed that IONM was independently and significantly related to RLNP with CD grade 2 or higher (odds ratio = 0.21, *P* = 0.02).

**Table 5:** Outcomes of univariate and multivariate analysis of the risk factors for recurrent laryngeal nerves palsy

	Univariate analysis			Multivariate analysis		
	Hazard ratio	95% CI	*P*‐value	Hazard ratio	95% CI	*P*‐value
Age, >70 y	0.60	0.18‐1.19	0.39	‐	‐	‐
Gender, Female	0.86	0.23‐3.24	0.83	‐	‐	‐
Brinkman index, >700	1.48	0.55‐3.98	0.42	‐	‐	‐
ICG, >10%	1.32	0.49‐3.53	0.57	‐	‐	‐
FEV1.0%, >70	0.99	0.36‐2.73	0.99	‐	‐	‐
DM	2.04	0.25‐16.4	0.50	‐	‐	‐
Prior therapy	0.87	0.32‐2.35	0.79	‐	‐	‐
Location, U	1.90	0.66‐5.47	0.23	0.54	0.13‐2.23	0.39
cT, 2‐3^a^	0.66	0.24‐1.80	0.42	‐	‐	‐
cN, 1‐2^a^	0.84	0.31‐2.30	0.74	‐	‐	‐
cRLN LN	1.07	0.36‐3.21	0.89	1.06	0.26‐4.25	0.92
Thoracic surgery time, >300 min	1.06	0.40‐2.84	0.89	‐	‐	‐
operative bleeding, >280	0.60	0.18‐1.92	0.39	‐	‐	‐
LN dissection, D3	1.23	0.46‐3.29	0.67	0.79	0.16‐3.74	0.76
Surgeons' experience, <30	1.63	0.58‐4.58	0.35	2.47	0.81‐7.50	0.11
IONM	2.94	1.04‐8.62	0.05	4.32	1.32‐14.2	0.01

Abbreviations: 24hCCr, 24‐hour urine creatinine clearance; D3, mediastinal, perigastric and cervical lymphadenectomy; DM, diabetes mellitus; FEV1.0, percentage forced expiratory volume in one second; ICG15R, Indocyanine green retention rate after 15 min; IONM, intraoperative neuromonitoring; LN, lymph node; RLN, recurrent laryngeal nerve; U, upper third; VC, percentage vital capacity.

^a^ Union for International Cancer Control 8th edition.

* *P*‐value for multivariate logistic regression analysis; significance was set at a value lower than 0.05.

### 3.3 Propensity score matching

Propensity score matching was used to balance the clinical characteristics and potential confounders between these two groups. After propensity score matching, the demographic and clinical characteristics before treatment were adequately balanced between the 47 pairs in the IONM and no IONM groups: standardized difference < 0.100 (Table 1). There were significant differences between the patients in the no IONM and IONM groups in terms of blood loss (280 mL vs 180 mL, *P* < 0.01) and postoperative fasting period (8 days vs 8 days, *P* = 0.02) (Table 2). The number of CD grade 2 or higher RLNP cases were lower in the IONM group than in the no IONM group (6.0% vs 21.2%, *P* = 0.02) (Table 3). A larger proportion of patients in the IONM group recovered from RLNP within 6 months compared to the no IONM group (87.5% vs 20.0%, *P* < 0.01) (Table 4).

### 3.4 Evaluation of LOS in IONM

Table 6 shows the correlation between LOS and postoperative RLNP in all patients with IONM. The sensitivity and specificity of the absence of LOS on IONM for intact RLN was 90.1% (60/66) and 50% (9/18), respectively. The positive predictive value (PPV) was 60% (9/15). The false‐response rate (i.e. LOS absence with RLNP) was 13% (9/69). The false‐no response rate (i.e. LOS without RLNP) was 9.1% (6/60). Five cases of LOS during the cervical procedure and one case during thoracic procedure were observed.

**Table 6:** Results of IONM

IONM	RLN0050		Total
	No	Yes	
*LOS*			
Negative	60	9	69
Positive	6	9	15
Total	66	18	

Note

Sensitivity: 60/66 = 90.1%; Specificity: 9/18 = 50%; Positive predictive values: 9/15 = 60%; Negative predictive values: 60/69 = 86.9%.

Abbreviations: IONM, intraoperative neuromonitoring; LOS, loss of signal; RLNP, recurrent laryngeal nerve palsy.

The video review identified the site of RLN injury. The causes of injury in PPV were burns with an ultrasonic scalpel in two cases, gripping with forceps in one case, dissection with scissors in one case, over traction in three cases, and cervical vagus nerve taping in two cases.

**FIGURE 2:**
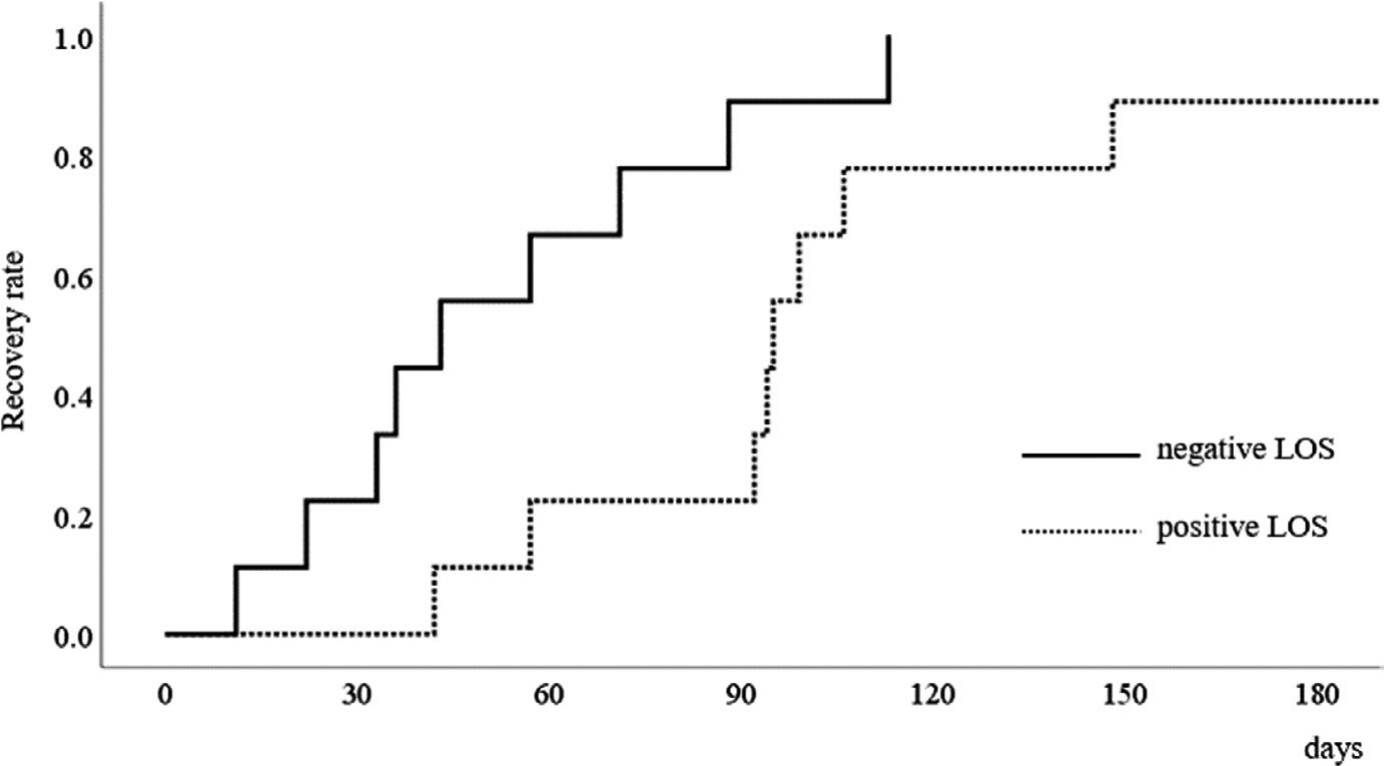
Kaplan–Meier curve of recovery from recurrent laryngeal nerve paralysis after esophagectomy. The recovery rate of the negative LOS group was higher than that of the positive LOS group, and the median durations to recovery were 43 d and 94 d, respectively. LOS, loss of signal

The Kaplan‐Meier curve of recovery from RLNP after esophagectomy in patients with IONM is shown in Figure 2. The time from RLNP to recovery was shorter without LOS than with LOS (*P* = 0.029). The median durations of recovery from RLNP were 43 and 95 days in the IONM and no IONM groups, respectively.

## 4 DISCUSSION

This study demonstrates the technical feasibility and reliability of intraoperative exploration, identification, and monitoring of the RLNs during thoracoscopic esophagectomy. Furthermore, our results suggest that IONM of RLN may reduce the severity of RLNP and aid in early recovery from postoperative vocal cord dysfunction.

In recent years, preoperative therapy has been the standard treatment strategy for advanced esophageal squamous cell carcinoma with lymph node metastasis.^8^ It has also been reported that the extent of mediastinal lymphadenectomy during esophagectomy in surgical treatment influences prognoses,^9^ particularly RLN lymph node dissection, which must be performed with extreme care in esophageal cancer surgery. A nationwide Japanese registry showed that the frequency of nodal metastasis is the highest in the upper mediastinal nodal station in patients with upper (42.9%) and middle (37.4%) esophageal cancers.^10^ Ninomiya et al reported that lymph node dissection by video‐assisted thoracoscopic (VATS) radical esophagectomy was beneficial in patients with lymph node metastasis around the bilateral RLNs.^11^ Therefore, complete dissection of the lymph nodes surrounding the RLN is recommended to decrease the rate of local recurrence and improve survival rates. Recently, the use of MIE has increased in esophageal cancer surgeries.^12^, ^13^ The RLN is very delicate and is easily damaged by traction and retraction as well as by direct sharp or thermal injury.

In a nationwide Japanese web‐based database, Takeuchi et al reported that the incidence of RLNP was higher in MIE than OE.^6^ RLNP causes not only voice hoarseness but also affects swallowing and is a risk factor for aspiration pneumonia. In severe cases such as those with bilateral nerve injury, tracheostomy may be required, which leads to great impairments in patients’ quality of life. Therefore, intraoperative measures to prevent RNLP are important.

IONM using EMG was developed and introduced in thyroid surgery^14^ with various studies showing that it contributes to a reduction in the rates of RLNP in such settings.^15^, ^16^ IONM during thyroid and parathyroid surgery has gained widespread acceptance as an adjunct to the gold standard of visual nerve identification.^17^, ^18^

While Hemmerling et al were the first to report the use of IONM in an esophageal surgery,^19^ several relevant reports have also been published in recent times.^20^, ^21^, ^22^, ^23^, ^24^, ^25^, ^26^ Hikage et al, who performed 56 procedures with IONM, reported on its feasibility and safety.^24^ Kobayashi et al demonstrated 31 cases where the rates of RLNP and aspiration were reduced by IONM.^26^ Zhong et al also indicated that the rates of postoperative RLNP and pneumonia reduced, and the quality of lymph node dissection improved with the use of IONM.^22^

In this study, IONM allowed surgery to be performed safely around the nerve, aided in the prevention of severe nerve conduction disorder, and reduced the rate of fatal nerve injury. The sensitivity of IONM in predicting paralysis reported in this study is comparable to those of previous reports. The false no‐response cases were observed in six patients in this series. Five cases of LOS detected during cervical procedures were observed on vagal stimulation before detachment around the RLN; this could be due to tube displacement due to change in body position. In addition, the muscle relaxants required for concurrent laparotomy may have affected the stimulatory response. In one case, during a thoracic procedure, LOS appeared after the lymph node dissection around the left recurrent laryngeal nerve. Although the position of the EMG tube was confirmed by an endoscope, there was no displacement, and a normal reaction could be observed by the stimulation of the right recurrent laryngeal nerve performed at the same time. Therefore, it is possible that transient neuropraxia appeared and was recovered from early after the operation. On the other hand, the PPV of IONM was low, and there were many false‐response cases. Randolph et al reported that false‐response (i.e. good EMG activity with postoperative vocal cord palsy) could result from a variety of situations, such as the following: (a) stimulation distal to the injured nerve segment; (b) injury after the last testing stimulation; (c) delayed neuropraxia due to progressive postoperative edema; (d) posterior branch injury with an intact anterior branch; (e) vocal cord immobility from non‐neural issues, such as laryngeal edema or arytenoid dislocation; and (f) EMG activity being initially present but its degree decreased significantly from the initial levels.^17^ The relationship between intraoperative nerve stimulation and postoperative volitional function is not completely understood, although it appears that an intense signal at the end of the surgery correlates very well with normal postoperative vocal cord function. Kanemura et al reported that the amplitude of the evoked response through stimulation was lower in recurrent laryngeal paralysis.^27^ Because we had defined LOS as a signal strength of less than 100 mV, there is a possibility that this increases the number of false‐responses. The severity of RLNP was CD grade 1 in seven patients and grade 2 in two patients, but no severe paralysis of grade 3 or higher was observed. The duration from paralysis to recovery was shorter in the false‐response cases than in true LOS cases. Seddon reported that peripheral nerve injury can be classified into neurotmesis, axonotmesis, and neurapraxia.^28^ Neurapraxia is unaccompanied by peripheral degeneration and recovers rapidly and completely. In false‐response cases, RLNP may have recovered early after a transient, delayed conduction disorder. The mechanism by which paralysis occurs could not be specified, but the duration to recovery from RLNP was thought to be short because of the minor degree of nerve damage.

It seems that the identification and verification of RLN using IONM could reduce the incidence of axonotmesis and neurotmesis during surgery. However, in intermittent IONM, it is necessary to confirm the stimulus response frequently in order to confirm functional preservation of the RLN. In the no IONM group, there was one case of intraoperative direct nerve injury, and in many cases, it was difficult to identify the site of injury. The low recovery rate in this group may be because the interruption of the axon or perineural nerves which led to the nerve palsy, did not recover due to misdirected innervation.

Recently, methods for the quantification of amplitude and latency for continuous monitoring have been reported, and these may be able to identify nerve damage more realistically and prevent nerve damage than intermittent methods.

Our findings show that intermittent IONM in MIE is easy to use and reduces the incidence of RLNP. This study has some limitations. It had a single‐center, retrospective design with a small sample size. A randomized controlled trial should be performed in such settings, as the details of minor surgical procedures change over time. In our study, grade 1 mild paralysis could not be prevented, and false‐response cases were observed. The newer real‐time continuously neural monitoring devices may be more useful for preventing nerve palsy.

In conclusion, IONM was useful in the identification and verification of recurrent nerves and could reduce the incidence of RLNP. We consider that IONM may increase the safety of esophagectomy and improve surgical outcomes.

## DISCLOSURES

Funding: No external funding was received for this study.

Conflict of Interest: Authors declare no conflicts of interest for this article.

Ethical Statement: The protocol for this study was approved by the Institutional Review Board for the Use of Human Subjects at Yamaguchi University School of Medicine (Approval No. H28‐181), and it conforms to the provisions of the Declaration of Helsinki. Written informed consent was obtained from all patients in the study.

## Supporting information

Video S1

Video S2
